# The Contrivance of Plant Growth Promoting Microbes to Mitigate Climate Change Impact in Agriculture

**DOI:** 10.3390/microorganisms9091841

**Published:** 2021-08-30

**Authors:** Angelika Fiodor, Surender Singh, Kumar Pranaw

**Affiliations:** 1 Department of Environmental Microbiology and Biotechnology, Institute of Microbiology, Faculty of Biology, University of Warsaw, Miecznikowa 1, 02-096 Warsaw, Poland; a.fiodor@uw.edu.pl; 2 Department of Microbiology, Central University of Haryana, Mahendergarh 123031, Haryana, India; ssriari@gmail.com

**Keywords:** abiotic stress, salinity stress, drought stress, plant-microbe interaction, sustainable agriculture

## Abstract

Combating the consequences of climate change is extremely important and critical in the context of feeding the world’s population. Crop simulation models have been extensively studied recently to investigate the impact of climate change on agricultural productivity and food security. Drought and salinity are major environmental stresses that cause changes in the physiological, biochemical, and molecular processes in plants, resulting in significant crop productivity losses. Excessive use of chemicals has become a severe threat to human health and the environment. The use of beneficial microorganisms is an environmentally friendly method of increasing crop yield under environmental stress conditions. These microbes enhance plant growth through various mechanisms such as production of hormones, ACC deaminase, VOCs and EPS, and modulate hormone synthesis and other metabolites in plants. This review aims to decipher the effect of plant growth promoting bacteria (PGPB) on plant health under abiotic soil stresses associated with global climate change (*viz*., drought and salinity). The application of stress-resistant PGPB may not only help in the combating the effects of abiotic stressors, but also lead to mitigation of climate change. More thorough molecular level studies are needed in the future to assess their cumulative influence on plant development.

## 1. Introduction

Soil, as an extremely heterogeneous environment, contains many different microorganisms with different properties. Plant health and soil fertility require balance and proper cooperation with beneficial microbes, especially bacteria [1]. In the natural environment, bacteria may occur freely in bulk soil from where they can be transferred to the rhizosphere, or internal parts of plants as endophytes [2,3]. The phytomicrobiome is a whole, well-structured community of all microorganisms in a given plant, associated with the host [2,3,4,5]. The rhizosphere, a thin film of soil around the roots, is the primary location of ion uptake for plants, simultaneously depositing nutrients and signaling molecules into this zone [6,7]. This special mixture secreted by plant roots contains low-molecular weight organic substances, such as carbohydrates, amino acids, fatty acids, organic acids, vitamins and a small amount of secondary metabolites [8,9]. The extremely carbon-rich root exudates create a unique space with maximum bacterial activity compared to the bulk soil [7,10]. The nature of secreting exudates and various genetic regulations can influence the structure of bacterial community in the soil [2]. This special recruitment of certain microbes enables the achievement of tangible benefits that would not be possible during solitary growth of the plant [11].

Climate change, despite its remarkable stability and repetition, is embedded in the Earth’s past. However, human actions are significantly accelerating these changes in global climate patterns [2,3]. The predominant signs of climate change are elevated mean surface temperature, ice melting, sea level rise and extreme weather events [3,4]. As a result of these rapid global alterations, crops are increasingly facing abiotic stresses, mainly drought, salinity and heat stress [2,9,12,13]. The demand for food is constantly increasing due to the growing population. Therefore, farmers have started using chemical pesticides and fertilizers in high doses to increase agricultural production [9,13]. Altered soil properties and structure due to climate change, along with artificial irrigation and unbalanced use of chemicals, are leading to the destruction of microbial communities in arable soils [11,14]. Since 1961, a nine-fold increase in fertilizer consumption has been observed worldwide [14]. The negative impact on the diversity of bacteria in the wheat rhizosphere under inorganic fertilizers has been established by Reid et al. [15]. According to model studies by Ortiz-Bobea et al., anthropogenic climate change has significantly affected global agricultural productivity [16]. The reduction in the amount of beneficial microorganisms and the disruption of nutrient cycling in the ecosystem has led to reduction in yields of about 21% since 1961. Nowadays, most farmers still use inorganic fertilizers, which creates a vicious cycle [16,17]. Additionally, the natural biocontrol properties of soil may become severely limited under these conditions, exposing plants to various diseases. Similarly, the host-pathogen relation may shift, as a result of global climate change [4].

Undoubtedly, the spread of land use nowadays is incomparably huge as opposed to the past. Three-quarters of non-ice land, that is, about 17 Mkm^2^, is actively utilized by humans, of which around 13% is cultivated, and the value is constantly growing. It has been estimated that since 1961 there has been a 3.5-fold increase of farmlands production and 2.5-fold increase of animal products [14]. As agriculture develops, the global food supply is rising, along with the growing trend of meat and vegetable oil consumption [8,14,18].

Climate change is affecting the health of crops around the world [19,20]. This phenomenon is expected to worsen in the future, and there is a clear need to address this situation. There is a large body of data in the literature on the effects of mitigation of abiotic stress by selected bacteria [5,21,22,23,24,25]. In the present review, an attempt is made to study the effects of using beneficial microorganisms, especially bacteria, on plant growth under major abiotic stresses (salinity and drought). We intend to show that certain microorganisms can help slow down these changes. In this literature review, various databases, such as PubMed (https://pubmed.ncbi.nlm.nih.gov/; accessed on 5 April 2021), EBSCO (https://www.ebsco.com/; accessed on 12 April 2021), SCOPUS (https://www.scopus.com/; accessed on 3 May 2021) and Google Scholar (https://scholar.google.com/; accessed on 7 May 2021), were searched for both quantitative and qualitative studies published in the last 5 years. The following search terms were applied: “abiotic stress”, “climate change”, “greenhouse gas emission” “pgpr” “drought”, “salinity” and their combinations using AND as a function word. A total of 2325 potentially relevant papers integrated were found, of which 220 were included in this review. Handsearching was also undertaken to include scientific papers with different key terms.

## 2. Climate Change and Its Impact on Agriculture

Climate change is a consequence of numerous phenomena, including fluctuations in solar radiation, shifts in earth’s movement in orbit, transformation of the atmosphere gasses composition, switches of the surface properties of the soil and ocean oscillations [23,26]. The last two mechanisms are influenced by both human activities and natural factors. The absence of global data and inability to assess the real impact of any given factor on climate change leads to constant scientific discussions among researchers and policy makers. 

Great emotions and controversies arise, above all, from the effects of climate change, which affects not only the ecological system but also human life [19]. Agriculture, as a sector highly dependent on weather conditions, soil quality and irrigation, is most vulnerable to climate change [27]. Droughts along with heat stress, floods, variability in rainfall and extreme weather events caused by climate change have major impacts on global agriculture [14,19,28]. As immobile organisms, plants are forced to adapt to a particular habitat in order to survive. Due to their adaptability in metabolism, physiology and development, they can undergo limited changes in response to various abiotic stresses [23,26]. Unfortunately, the effect of a particular stress on a plant may reduce its tolerance to another abiotic factor. This makes plants extremely sensitive [29]. Surprisingly, some plants under multiple abiotic stresses may develop specified modifications as a multivariate tolerance response.

### 2.1. Global Warming

Global warming is defined by the Intergovernmental Panel on Climate Change (IPCC) as the “rise in combined surface air and sea surface temperatures averaged throughout the world and over a 30-year period.” [30]. It is also stated that human activities have become the primary cause of change in the planet as it transitions from the relatively stable Holocene to a new era known as the Anthropocene. According to the research, human-induced global warming has reached about 1 °C above pre-industrial levels. However, in many regions a much higher temperature increase than the global average has been observed. According to the projections, this value may increase up to 1.5 °C or even 4.8 °C in 2100 [14,31]. Moreover, the warming over land is faster than the global average. The mean temperature over land in the period 2006–2015 was 1.53 higher than in 1850–1900 [14].

Temperature is a key ecological factor with a direct impact on physiological processes and plant development. An increase in temperature has a positive effect on faster development of green mass and a shorter duration of cultivation [32]. However, a sharp drop or rise in temperature damages the plant cells, which ultimately leads in an overall lower yield [32,33]. Zhao et al. have reported that yield losses in maize, wheat, rice and soybean crops ranged from 3.1% to 7.4% per one degree Celsius increase in global mean temperature [32]. Other studies found a 6% and 4% decline in wheat and maize yields, respectively, over a 29-year period of warming trends [34]. Heat stress due to increased temperature is a limiting factor on photosynthesis, especially for C_3_ crops such as rice and wheat, but also for C_4_ plants, maize and sugarcane crops [32,34,35,36]. In addition, temperature increase affects plants through changes in humidity. Lowering the water vapor content in the air leads to water loss from the plant, causing stomata to close and reducing the efficiency of photosynthesis [37,38]. Prolonged high temperatures lead to drought and water stress in plants, which in turn leads to water scarcity [38]. 

The change in global average temperature is not the only threat posed by global warming to crops worldwide [39]. Other serious menaces are sea level rise, desertification, altered precipitation and extreme weather events. Sea level rise leads to land loss and salinization of freshwater due to water mixing. As the hydrological cycle intensifies with global warming, exacerbation of unusual weather events, such as extreme precipitation, flooding and heat waves, has been observed [17,40,41,42]. Global warming may also have many unforeseen effects. Recent observations show that the melting of the ice sheet on Greenland may become a source of highly toxic mercury pollution in Atlantic waters [43].

### 2.2. Greenhouse Gas Emission

Greenhouse gases (GHGs) are components of the Earth’s troposphere, which can retain solar energy, due to their physicochemical properties. The presence of these gaseous constituents in the atmosphere retains an average temperature of 14 °C, which allows plants to grow freely in most lands. Their absence would leave the temperature around -19 °C, making life on Earth impossible. The major environmental sources of greenhouse gases emission are forest fires, wetlands, oceans, permafrost, volcanoes and earthquakes [44]. They are usually naturally absorbed by the environment. However, the surplus caused by human activity disturbs this balance [44]. The most prevalent GHGs produced by anthropogenic processes are carbon dioxide (CO_2_), methane (CH_4_) and nitrous oxide (N_2_O). It is estimated that it is the emission of these gases that contributes to over 90% of anthropogenic climate warming. In addition, sulfur dioxide (SO_2_), hydrofluorocarbons (HFCs), perfluorocarbons (PFCs) and sulfur hexafluoride (SF6) are produced [45].

The intensification of global warming processes has been noted along with the increase of GHG release [46]. The ever-growing human population has accelerated the demand for energy, as a result of industrialization, urbanization and globalization [23,43]. Comparing the CO_2_ content in the air before the industrial revolution with the present condition, it is clearly shown that humans are the main contributors in the formation of this gas [23,43]. CO_2_ concentration is rapidly increasing, growing from 280 ppm in pre-industrial times to 400 ppm today. The forecast for year 2100 gives 540 ppm, and in the high emission scenario even 940 ppm [28]. The increase in land use due to deforestation contributes to a quarter of the increase in total CO_2_ emissions [14]. Fossil carbon, which has formed in the Earth’s crust over millions of years, is released into the atmosphere as CO_2_ in enormous quantities, over the course of decades [47]. 

In 2018–2019, GHG emissions in the United States of America and UE reached 6.5 and 4.4 billion metric tons of CO_2_ equivalent, respectively [48,49]. During 2014, in the list of the world’s topmost polluters, China was on top with 30% of all CO_2_ emissions, whereas others such as the US, EU, India, Russia and Japan contributed 15%, 9%, 7%, 5% and 4% of CO2 emissions, respectively [49]. The global GHG emissions can be evaluated on the basis of different major economic sectors, such as (i) electricity and heat production; (ii) agriculture, forestry and other land use; (iii) industry; (iv) transportation; (v) building; and (vi) other industries. Agriculture, forestry and other land use accounted for 24% of 2010 global greenhouse gas emissions [49]. Agriculture alone had a proportion that fluctuated between 1 and 14 percent, which is comparable to industry. Agriculture has increased its GHG emissions by 12% since 1990. This is a result of a 9% rise in N_2_O emissions from soil management practices, as well as a 60% rise in combined CH_4_ and N_2_O emissions from animal manure management systems, indicating the growing usage of emission-intensive liquid systems over time. Various agricultural soil management strategies can improve nitrogen availability in the soil and result in nitrous oxide emissions (N_2_O). The use of synthetic and organic fertilizers, the growth of nitrogen-fixing crops, the drainage of organic soils and irrigation methods are all activities that contribute to N_2_O emissions from agricultural areas. Agricultural soil management is responsible for just over half of the GHG emissions generated by the agriculture economic sector [49]. Fertilizing crops with the correct quantity of nitrogen for optimal crop production can help in reducing emission of GHGs, since excessive nitrogen application can result in increased nitrous oxide emission without improving crop output.

GHG emissions can affect agriculture through changes in photosynthetic rates and nutrient losses in plants [28]. Elevated CO_2_ concentrations increase photosynthesis and water use efficiency, leading to reduced transpiration. However, these processes only slightly mitigate the reduced yields caused by global warming. Rapid plant growth under high CO_2_ concentrations alters the content of elements that are essential for humans and animals. Studies have shown that cereals and legumes growing under a CO_2_ concentration of 550 ppm have 3–11% lower levels of zinc and iron [45,46]. Shifting the concentration to 690 ppm resulted in decrease in mineral content in plants to about 5–13% of phosphorus, potassium, calcium, sulfur, magnesium, iron, zinc, and copper concentration in various crops [50]. Decline in protein and nitrogen content in edible parts of C_3_ plants under elevated CO_2_ has also been observed [22]. Therefore, a steady increase in the concentration of this gas may exacerbate the nutritional problems of people in poor parts of the world, due to nutrient losses and lower yields [50].

### 2.3. Abiotic Stresses

Climate change leads to various abiotic stresses that affect agriculture [26,51]. Raised temperature may lead to drought and heat stress, while variability in precipitation can cause extreme weather events and flooding [8,41,52,53]. Another type of abiotic stress, salinity stress, is the result of several factors [8,54,55]. Plant response to abiotic stress is mainly determined by the type of stress, exposure time, plant species and health status. Moreover, plants under combined stresses show lower tolerance to other factors [42]. Particular stresses of high intensity, such as heat, can be harmful, even they are of short duration. Mineral deficiency, on the other hand, can take months to become stressful. Natural mechanisms for stress mitigation that plants employ include reducing organ growth rate, closing stomata, lowering their photosynthesis rate or stimulating root growth [36,42]. There are many studies on changes in transcriptome under abiotic stress. For instance, Cohen and Leach reported that drought stress in rice leads to changes in the transcription of 201 core genes [42].

#### 2.3.1. Salinization

Soil salinization, a multifactorial and complex phenomenon, is gradually affecting more and more arable land. This excessive accumulation of mineral salts can develop in various ways, regardless of latitude and climatic zones [9]. Arable soils that are particularly affected by salinization process are located in arid and semiarid regions, where precipitation is insufficient and the evapotranspiration rate is quite high [14,49]. Soil is considered as salinity-affected when the salt concentration in the soil solution reaches 3 to 5 g per liter and the electrolytic conductivity parameter is 2 to 4 or above 4 mSm/cm (25 °C) [56]. Primary soil salinization results from a natural salt accumulation process. This is mainly associated with drought and extreme weather conditions. Sometimes, high salinity is the result of a low water table, especially if the area is regularly flooded by saline water. Secondary salinity is caused by anthropogenic activities, due to excessive irrigation and leaching of soils in the absence of proper drainage systems [8,56,57,58]. The reason for high salinity may also be the use of highly mineralized irrigation water and massive fertilization [59]. Agriculture worldwide has always faced the problem of soil salinization [14,52]. Salinization is dynamic and spreading in almost 100 countries with an annual growth rate of about 1–2% [9]. The latest available data have demonstrated that salinity, together with sodicity, has affected around 1030 million hectares (412 million hectares of saline soils and 618 million hectares of sodic soils) of global land [60]. Initial estimates suggest that nearly one-third to even 50% of irrigated land is salt-affected, making it unsuitable for cultivation [9]. In populous countries such as India, salinity is considered one of the greatest threats to national food security and economic development [8]. 

Highly harmful salinity stress affects almost all aspects of the physiology and biochemistry of plants, and therefore contributes to a marked decrease in crop yield and productivity [61]. High salinity is manifested not only by a decrease in dry mass, biomass accumulation and overall water content, but also in an impaired root system, low yield, low chlorophyll content and low seed germination rate [52,55,62,63,64]. The negative effects of salinity on crops may result from the indirect action of salts in the cultivated soil or from direct harmful effects on plants. The accumulation of salt in the soil adversely affects its physicochemical properties, which increase the dispersion and swelling capacity, and thus reducing the soil water permeability [65]. Such soils gradually become poorer in nutrients and experience reduced microbial activity, which results in a decrease in biomass and organic matter production. Land, as mentioned above, also loses its buffer and antipollution properties [56]. The long-term salinization process, without any human intervention, results in a drastic decrease in soil fertility, and thus its uselessness [66]. Increased salt levels not only damage soil structure and microbial activity, but they also limit plant development by generating osmotic impact, hazardous ion and mineral imbalances, or metabolic diseases [51,67]. Osmotic stress occurs as a result of the excessive concentration of salts, decreased water potential and plants’ inability to uptake water. Consequently, this leads to an ion imbalance in the plants and accumulation of salts ions in plant tissues [68,69]. This can contribute to heavy damage to plant metabolisms, which can further lead to stunted plant growth and reduced yield [65].

Inadequate pH of soil, both very low and very high, worsens the bioavailability of elements from the soil due to their presence in the soil in water-insoluble forms [61]. Moreover, under harsh conditions, plants need additional nutrients to cope with salinity stress [13]. Inaccessibility of essential ions generates several harmful effects on plants, manifested mainly by leaf chlorosis, halting increases of shoots and roots or flowering disorders. Prolonged deficiency of required elements displays dieback of root and shoots, followed by whole-plant death [67].

Too high a concentration of Na^+^ ions in the roots causes not only osmotic stress, but also has a negative impact on the transport of K^+^ ions to plant cells. Moreover, a very high Na + ion concentration in plant cells results in various physiological disorders, such as reduced flowering or fruiting [13]. The following metabolic processes are very sensitive to increased salinity: transport of electrons, phosphorylation, photosynthesis and photorespiration [48,66]. Salt stress significantly lowers the efficiency of photosynthesis due to its multi-level action. The uptake and accumulation of Na^+^ and Cl^-^ can act as photosynthesis inhibitors which disrupt photosynthesis and reduce the production and size of leaves, which can lead to plant death [13,70]. Nevertheless, the toxic effect of salts is less harmful than osmotic stress. The higher concentration of ions in the environment is accompanied by their more intensive uptake by plants. This slightly reduces the water potential of the roots and, as a result, stimulates the water uptake by the plant [30,54].

There are several critical moments in a plant’s life which determine its vulnerability to stresses, including high salinity. The most sensitive stages are seed germination, early seedling growth, fertilization and pollen development [28,56]. Many studies have proven that a high salt concentration significantly reduces the ability of seeds to germinate in soybean, sunflower, pepper and maize [71,72,73,74]. Additionally, it has been reported that high salinity inhibited post-germinative growth in soybean, pepper and maize [73,74]. However, the subsequent stages of plant development in particular plants, such as peanut, could be sensitive to excessive salinity [19,57].

Phytohormones are exceptional plant regulators and metabolic activity coordinators, closely involved in growth and development [7,13]. These organic substances can act as signaling molecules in response to various environmental factors [1]. For instance, auxin stimulates the growth of adventitious roots, and cytokinin enhances cell proliferation and delays the plant-aging processes [75,76]. Under stress conditions, poor secretion or lack of hormones can slow down plant growth. In contrast, supplementing of hormones can improve stress tolerance [13,77].

Plants under salinity stress tend to increase the production of the hormone ABA and ethylene, in contrast to IAA, cytokinins, salicylic acid (SA) and jasmonic acid (JA), whose production is reduced [6]. Stress-derived ABA tends to decrease the water stress under high salinity by closing the stomatal apparatus and providing osmotic adjustment [6]. This helps in reducing water loss due to low transpiration. However, stomata that are closed for too long reduce photosynthetic activity and accelerate aging [78]. GA plays an important role in regulating important processes in the overall plant development, including seed germination, stem elongation and flowering [10]. Auxins can initiate root growth, postpone leaf senescence and promote stem elongation under normal conditions [6], while cytokinin improves cell proliferation and enlargement and the division of chloroplasts, and simultaneously reduces stomatal closure and delay leaf senescence. SA and JA have also been reported to be involved in various plant defense responses to alleviate salinity stress [6,7,78]. The reduction of photosynthesis efficiency at high salinity may occur as a result of damage to photosystem II (PSII) [74]. Insufficient water content may lead to inhibition of photosynthesis due to a lack of substrate during the light-dependent reactions. Additionally, lack of water, which is a natural environment for most metabolic reactions, may reduce photosynthesis [79,80].

High salinity, similar to drought conditions, creates oxidative stress. This usually leads to the formation of reactive oxygen species (ROS) as a result of different changes in the physiology and metabolism of plants. Under unfavorable conditions, ROS concentration increases to counteract the effects of stress. These signaling molecules may regulate the process of programmed cell death, closing the stomata or defending plants against pathogens [21,49,67]. However, the disturbance of cell homeostasis due to ion imbalance generates ROS [81,82]. Excessive ROS accumulation in plant cells leads to numerous changes and damages, which in turn affect the condition of the plant, its yield and survival [83]. This could affect DNA, chlorophyll, enzymes and other proteins, which may lead to detrimental effects, and finally even cell death [20,84,85,86]. To mitigate damage under this stress, plants have created complex antioxidative defense systems, in which involved are antioxidative enzymes and other non-enzymatic antioxidant mechanisms [6]. Superoxidase dismutase (SOD, EC 1.15.1.1) is mainly involved as O_2_^•−^ scavenger, which generates H_2_O_2_ and O_2_. Therefore, H_2_O_2_ can be scavenged by catalase (CAT EC 1.11.1.6), ascorbate peroxidase (APX, EC 1.11.1.11) and guaiacol peroxidase (GPX, EC 1.11.1.7) [65,87]. It has been reported that the increase in ABA production in plants under salinity stress induces antioxidant defense genes such as APX, SOD or CAT to remove ROS molecules [85]. Decreased activity of SOD, GPX, APX and CAT activity in roots and also CAT in leaves of a salt-sensitive maize genotype has been demonstrated, while a salt-tolerant maize genotype had increased SOD activity in leaves. Further, glutathione reductase (GR) may take a role in redox balance maintaining due to reduced glutathione (GSH) restoration [88]. In addition, non-enzymatic molecules take part in damage prevention against ROS, such as glycine betaine or carotenoids [65,89].

Malondialdehyde (MDA) is the main product of polyunsaturated fatty acids peroxidation, and thus is used as a biomarker of oxidative stress [24,73,90]. It has been demonstrated increased level of MDA under salinity conditions in various crops [6,90]. MDA has high biological activity and the ability to move over long distances, so it can act far away from the site of its origin. This organic compound tends to inactivate enzymes, and indirectly affects the processes of protein synthesis [85].

#### 2.3.2. Alkalinity and Acidity

Soil pH creates suitable conditions for microorganisms and plants [91,92]. The final soil pH depends on many factors, the most important of which are precipitation and evaporation [93]. A relatively high H^+^ content in relation to OH^-^ leads to soil acidification, while a relatively low H^+^ content leads to its alkalinity. Alkaline stress is a condition where an abnormally high soil pH has occurred [94], whereas a soil is considered acidic when the pH is below 5.5–5.0, which leads to acidic stress in plants [95]. Areas that currently have problems with alkaline and acidic soils include China, Europe and Africa [95]. Only 4.5% of acidic soils are used in agriculture. In contrast, about half of salt-affected lands are alkaline [95].

Extremes of pH negatively affect the physico-chemical properties and many biogeochemical processes of the soil, thus deteriorating the condition of crops and reducing yield, which is why they are referred to as stresses [75]. Soil pH strongly has a great influence on solubility of nutrients, and the optimum pH for the uptake of elements is slightly acidic. Therefore, both too acidic and too alkaline conditions are unfavorable for crop production as they affect the uptake of elements [76]. Long-term stress leads to a deficiency of nutrients that are essential for plant development and metabolic processes [95]. In addition, H^+^ concentration strongly influences soil structure and groundwater potential [93]. As noticed earlier, combined abiotic stresses can have a stronger negative effect on the plant. Alkaline stress under salinity has been shown to be more detrimental than salinity stress. Plants exposed to both acidic and alkaline environments may experience numerous damages. Roots can suffer damage to epidermal and cortical cells, depolarization of membranes, swollen root hairs or cracks in root meristem regions [95]. Apart from the apparent consequences for agriculture, the overall impact of acidity and alkalinity on crop health condition is underestimated. For this reason, measures to combat this stress in particular are limited [82]. 

#### 2.3.3. Drought

Climate change is increasingly causing unexpected periods of water scarcity in habitats that rapidly accelerate and disrupt hydrological processes. Drought as an extreme threat to the environment can occur naturally or as a result of anthropogenic activities [41,96]. In relation to the area, the following types of droughts are distinguished: hydrological, meteorological and agricultural. However, since they are closely related, it is difficult to separate them [96]. Water shortage on agricultural land may be a consequence of meteorological drought, due to low precipitation. Moreover, it may be related to hydrological drought due to insufficient amount of groundwater [73,97]. It is estimated that about 60% of total agricultural and livestock sectors is already affected by desertification [27,74]. Apart from water scarcity, drought leads to other harmful consequences such as increased risk of wildfire, loss of crops and livestock, or indirect health effects on people [77].

Water deficiency in crops is mainly due to the natural effect of drought or osmotic stress in high salinity, acidity or alkalinity [25,98]. The rate and duration of water shortage are critical for plant adaptation and maintenance. [25]. Climate change, leading to a continuous raise in the average temperature on Earth, is reducing water resources, resulting in an increasing expansion of areas suffering from drought. Farmers’ struggle to combat drought with fertilizers and irrigation only exacerbates the problem in the long run [2,27,98]. A prominent example is the disappearance of the Aral Sea, one of the largest inland seas in the world. As a result of irrigation of large arid areas for cotton cultivation in Central Asia, the inflow of water into the Aral Sea has gradually declined. Only a few smaller reservoirs remain, with salinities higher than those of the original ones [27]. 

Water uptake by a plant occurs vertically upward through the steam as a result of the water potential gradient between the soil and the plant and within the plant. Lack of water in the soil leads to a decrease in the water potential in the soil and thus to a decrease in water uptake [70]. Thus, drought conditions generate information that is transmitted to the plant’s leaves via ABA and other hydraulic signals. As a result, the plant protects itself against these conditions through a number of processes, such as closing stomata [17,70]. This is to prevent the water potential inside the plant from being reduced, which would only exacerbate the growing problem. One of the measures of soil water potential reduction is stomatal conductance [70]. Soil water availability can be expressed as soil water content or soil water potential. While soil water content is the amount of water present, water potential is the amount of water available to the plant [99,100]. Thresholds of available soil water to plants vary widely and depend on atmospheric conditions, soil type and plant species [100].

The water requirements of plants depend on their stage of development. However some of them are particularly susceptible to water shortage, such as the grain-filling period or flowering [20,25]. Soil water shortage usually inhibits proper growth and development. Less access to water increases the concentration of ions. Both water scarcity and high salinity can create hypertonic conditions leading to osmotic stress, thus disturbing the nutrient and water balance, the permeability of membranes, and reducing the activity of selected enzymes [61].

ABA, the so-called stress hormone, is a key molecule in both drought and salinity stress. It is one of the main regulators of drought mitigation processes [101,102]. The level of the hormone correlates closely with the degree of stress, so it is a good measure of it [102]. The presence of the soluble receptor ABA in plants is unique. The increase in the level of the hormone initiates the cellular response to this abiotic stress. As a result, the plant closes stomata and changes the expression level of stress-related genes. Thus, the plant tries to adapt to the new conditions. ABA plays an important role in changing the growth ratio of shoot growth to root growth. In addition, it supports root length growth. All these mechanisms contribute to increased water uptake by the plant [101,102].

Osmoprotectants (osmolytes), extracellular polymeric substances and volatile organic compounds (VOCs) are the best known and are valued for their special properties. They can increase the survival of microorganisms, but also of plants under various stress conditions, especially in high salinity [21,49,52,103]. The accumulation of osmoprotectants, electrically neutral, non-toxic and low molecular weight metabolites, reduces osmotic stress. Their effectiveness is based on supporting of turgor pressure in cells and ion transport across the plasma membrane [51,61,104]. Proline is a particularly important osmoprotectant for plants under drought conditions. The increase in its concentration is a marker of abiotic stress caused by water scarcity [105,106,107]. Proline accumulation in the cell allows reduction of the osmotic stress caused by the reduced amount of water. This gives the plant a longer time to survive a dry period. It also protects against numerous damages caused by abiotic stress. Proline reduces water potential and protects cells from the toxic effects of accumulated ions. Plant mutants that cannot produce proline are significantly less drought tolerant [106,107].

Cell growth and photosynthesis are the most sensitive processes under water deficit conditions [37,108]. Maintaining optimal turgor of leaf cells is critical in the proper performance of photosynthesis, resulting in high photosynthetic rates. For intact and proper functioning of the plant, undisturbed photosynthesis is required, which is a key process that provides energy compounds and substrates for plant development. Photosynthesis can be restricted by closed stomata, which prevents CO_2_ capture and thus transport of this gas by leaf mesophyll [2,35,108]. Regulation of stomatal opening ensures proper hydration of the plant, as well as water uptake from the soil [109]. In addition, water deficiency can reduce chlorophyll content or cause metabolic disorders [25]. Roots, as the main water uptake organs, play a crucial role in the survival of plants [110]. Therefore, the increased absorption surface of the root can compensate or partially mitigate the negative effects resulting from reduced water supply. There are few hormones whose increased production is observed in conditions of water shortage, such as ethylene, ABA and auxins. Water scarcity decreases rates of photosynthetic CO_2_ assimilation due to stomatal closure [111,112].

Higher concentrations of cellular components and molecules in cells reduce viscosity. These situations endanger the plant by lowering enzymatic activity and decreasing water flux from xylem to the cells. This may inhibit mitosis, preventing the plant from growing [20]. Severe dehydration induces photorespiration as a result of both low CO_2_ concentration and the unavailability of a sufficient amount of water. Drought, similar to salinity stress, leads to higher production of ROS, resulting in oxidative stress [88]. Certain antioxidant enzymes, such as CAT and APX, seem to play a special role in plant tolerance to drought stress [113]. Prolonged drought can impair photosynthesis due to a reduced amount of chlorophyll, which in turn reduces plant growth and yield. Low concentrations of ethylene have a positive effect on adventitious root development and fruit ripening, while high production of ethylene under abiotic stress conditions, including drought, leads to many harmful effects, such as inhibition of root and shoot growth, defoliation and premature senescence [6,114].

Drought tolerance is usually the result of many biochemical and physiological adaptations, which consequently allow the plant to maintain its desired size and yield despite unfavorable environmental conditions. However, in the case of long-term or sudden changes, the plant is unable to cope and needs external help to survive.

## 3. Plant Growth Promoting Bacteria (PGPB)

### 3.1. About PGPB

A group of bacteria that help promote plant growth thanks to their unique properties is called plant growth promoting bacteria (PGPB). PGPB isolated only from the root zone, on the other hand, are called plant growth promoting rhizobacteria (PGPR) [18,21]. It has been estimated that only 2 to 5% of rhizosphere bacteria exhibit PGPR properties (Table 1) [115]. PGPB can be used effectively under conditions of nutrient deficiency and are gradually replacing fertilizers. As phytostimulants, PGPB can increase plant growth and crop yield. Some of these bacteria can suppress phytopathogens by producing various metabolites, which is referred to biocontrol properties [1]. The use of microorganisms instead of synthetic chemicals provides a non-hazardous approach to plant health that increases agricultural productivity and can limit negative effects of disease [3,21,59,85,86,92]. Despite numerous benefits, the use of PGPB in agriculture is currently not the leading trend. Many factors are to blame for this, which will be discussed later in this paper [1].

The beneficial effects on the plant through interaction with PGPB can be achieved both directly as well as indirectly [10,79]. Direct mechanisms are based on the production of plant growth promoting substances or biofertilization by mobilizing mineral soil components [115]. These processes have a decisive influence on the condition of the plant and their development [6,9,80,81,82]. Reducing the impact of plant diseases caused by pathogens, mitigating abiotic stress or inducing systemic resistance in competition for nutrients and niches are categorised as indirect mechanisms [6,83]. The most common and successful PGPB belong mainly to the genera *Agrobacterium*, *Azospirillum*, *Azotobacter*, *Bacillus*, *Burkholderia*, *Pseudomonas*, *Streptomyces* and *Serratia* [52,84].

Elements such as nitrogen or phosphorous are the most limiting nutrients in crop production [6,50]. Element fixation, solubilization and mineralization are key processes by which microbes can enrich the soil with nutrients that can be easily taken up by plants [1,103]. Fixing nitrogen from the atmosphere into organic forms allows plants to assimilate these compounds. Some of the PGPB can solubilize phosphate, potassium and zinc salts [104,116,117,118,119,120]. In addition, the activity of some PGPB leads to an increase in root surface area and root morphology, which improves nutrient uptake [121]. Furthermore, PGPB can influence ion uptake by altering the transcript levels of selected genes involved in this process. A strain of *Achromobacter* isolated from *Brassica napus* roots enhanced the rate of nitrate (NO_3_^-^) uptake by *B. napus*. Further, Calvo et al. showed higher transcript levels of five nitrate and four genes responsible for ammonium uptake in *Arabidopsis thaliana* treated with *Bacillus* sp. [122]. 

Phytohormones produced by bacteria can act as plant growth regulators involved in plant development, physiology and immunity [123]. PGPB can synthesize auxins, gibberellins, cytokinin and abscisic acid [2,99,100,108,115]. Auxins, especially indole-3-acetic acid (IAA) produced by PGPB, can stimulate root growth, nodulation and cell proliferation [7,56]. Synthesis of auxin has been demonstrated several times by different strains, mainly represented by the genera: *Bacillus*, *Burkholderia*, *Serratia*, *Aeromonas* and *Azospirillum* [115]. Some PGPB are also cytokinin and gibberellin producers, but further research is needed to determine the role of these bacterial hormones in plant growth [2,13,124]. It has been presented that *Azospirillum* strains producing gibberellin resulted in growth promotion when inoculated into maize roots [125]. In addition, Parmar et al. demonstrated the ability to produce gibberellin in fluorescent *Pseudomonas* strains [124].

There are already many bacterial biofertilizers in commercial use [3]. The most common are based on nitrogen-fixing microbes, such as Nitragin Gold^®^ containing *Rhizobia*, produced in the USA [126]; Nodulest 10 with *Bradyrhizobium japonicum,* from Argentina, or Azo-N with *Azospirillum brasilense* and *Azospirillum lipoferum,* from South Africa [3,127]. Some commercial biofertilizers are based on phosphate solubilizers, such as Phosphobacterin with *Bacillus megaterium* from Russia, or potassium solubilizers, such as K Sol B with *Frateuria aurantia*, from India [3]. In addition, a few are phytostimulators, such as Amase^®^ with *Pseudomonas azotoformans*, and biocontrol agents, such as Cerall^®^ with *Pseudomonas chlororaphis*, both from Sweden [3,127].

There are several methods of PGPB application to plants. Some of the most commonly used are carrier-based inoculation, direct soil application and seed treatment. Another less common method is root dipping in the bacterial suspension or application of bacteria through drip irrigation [128]. Application to the soil requires a carrier that strongly supports the survival and colonization ability of the microbe in the rhizosphere or plant [128]. Selection of individual bacteria for inoculum should be based on expected characteristics, environmental requirements and possible interactions with the host plant and other soil microorganisms [129]. It is highly recommended to use autochthonous microorganisms that already have the ability to live in a particular environment, including climate and soil type [130]. The immune system of plants is another point to consider. Plants have multiple defense barriers and can recognize potential aggressors through pattern recognition receptors [129]. PGPB candidates should be competitive enough to successfully colonize plants. Microbial consortia, as a group of species acting together as a community, improve and maintain the soil physical and chemical properties [129]. Some of the PGPB act synergistically when applied together or coexist, such as *B. subtilis* with mycorrhizal fungi [9,19]. Gomez-Godinez et al. revealed a stronger effect on maize when using multispecies inoculum of PGPB than single bacteria [131]. Inoculation with a single microbial culture may place the isolates in a worst position, because may suffer from competitive and deleterious effects of local soil microorganisms [108,132].

The biocontrol properties of selected PGPB reduce the survival of phytopathogens and thus protect plants from certain pathogens. Biocontrol properties are the result of many different adaptations to the environment. They may synthesise different hydrolytic enzymes such as cellulases, proteases and β-glucanases [105,113]. In addition to these enzymes, they can produce many other substances such as siderophores, hormones, HCN and various volatile organic compounds [125]. Studies have showed that *B. altitunidis* KP-14 had antagonistic activity against both fungi *Fusarium culmorum* and *Botrytis cinerea*, and also *Bacillus* sp. KP-18 against *F. culmorum* [133]. Cytokinin secreted by *Pseudomonas fluorescens* G20-18 is an important biocontrol agent against the pathogenetic *Pseudomonas syringae* for *A. thaliana* [123]. Selected strains of *Bacillus subtilis* have antifungal activity against *Fusarium oxysporium* and *Rosellinia necatrix* due to antibiotic production [134]. Antagonism of *Bacillus amyloliquefaciens* against *Fusarium oxysporum* has also been reported [117].

### 3.2. Abiotic Stress Tolerant PGPB

Abiotic stresses, as mentioned earlier, adversely impact on physical-chemical soil properties and microbial communities [23,103,135]. As a selection factor, long-term abiotic stress contributes to the evolution of specific microorganisms, able to survive in adverse environmental conditions [23,104]. Bacteria belonging to particular species differ in stress tolerance. Although some species do well with abiotic stresses, because of individual properties, only selected strains are able to live under particular conditions [136,137]. Some of the PGPB are able to survive and proliferate under stressful conditions due to different adaptation mechanisms [13]. In this work, we refer to these microbes as abiotic stress tolerant growth promoting bacteria (AST-PGPB). Normally, microorganisms require a longer period of adaptation under rapid environmental changes when interacting with the host plant. The composition of root exudates tends to change under particular stressful conditions. Synthesis of particular substances can stimulate the mechanisms of counteracting abiotic stress in microorganisms [136,138]. Therefore, selected biochemical compounds may participate in the close interaction between microbes and plants by activating specific microbial stress genes. Such plant-associated microorganisms generally adapt much faster to new stress conditions, which supports microbial survival. This unusual relation makes AST-PGB an efficient tool for promoting plant growth under abiotic stress conditions [18,135]. It has been reported that some AST-PGPB are even more active under harsh environmental conditions [52,84]. Nagaraju et al. demonstrated that the solubilization of zinc compounds decreases significantly with increasing salinity [139]. In addition, selected AST-PGPB may not exhibit PGPB properties or promote plant growth under standard conditions. However, when used under harsh conditions, they may exhibit growth-promoting and stress-alleviating effects [18,52,61].

Selected AST-PGPB have evolved different adaptation mechanisms to specific abiotic stresses or multi-stress conditions in the soil (Table 1). Thanks to plant growth promoting properties, these microbes can help plants tremendously to cope with the effects of abiotic stress [53]. The ability of AST-PGPB to produce specific secondary metabolites to combat the effects of abiotic stress is increasingly appreciated due to its beneficial effects on crops [67]. Osmoprotectants, as highly soluble organic substances, can be produced by the microorganisms and released into the external environment [13]. Some plants may lack the machinery to produce certain osmolytes, so external additions using microbes are highly desirable. AST-PGPB can accumulate proline, glycine-betaine, glutamate and trehalose, which can help them to cope with osmotic stress [21,117,136]. It was found that the survival of *Pseudomonas aeruginosa* GG RJ21 under drought stress conditions was possible due to mass production of glycine-betaine and proline [109], while *Azospirillum* spp. strains can also accumulate proline, trehalose and glycine-betaine. In addition, proline has osmolytic properties and also acts as a ROS scavenger involved in protein stabilization [67]. The production of extracellular polymeric substances is an important criterion for the selection of candidates for AST-PGPB [13]. Exopolysaccharide (EPS) secretion allows bacteria to protect themselves from harsh environmental conditions through their bacterial biofilm. EPS can mobilize certain ions, store nutrients, participate in the formation of microconsortia and promote colonization of root surfaces [140,141]. Bacteria are able to tolerate wide fluctuations of various environmental factors due to stabilizing the cell membrane against external factors through EPS [125]. It was shown that strain mutants of *P. aeruginosa* exhibited an 86% reduction in EPS lost tolerance to high salinity compared to control strain *P. aeruginosa* PF23 [142]. 

Some of AST-PGPB bacteria exposed to abiotic stress conditions are capable of producing VOCs. These lipophilic, low molecular weight compounds are often produced by microbes as regulators of various properties [56,143]. Plants can use these substances as indicators, by which they recognize microbial species of with which they interact profitably [67]. Mainly, the production of acetoin, butanediol, 1,3-propanediol, geosmin and dimethyl disulfide by bacteria have been reported [67,117]. However it is estimated that thousands of such compounds are produced by different bacteria, such as alcohols, alkanes, alkenes, aldehydes, esters, ketones, organic acids or sulfur compounds [144].

## 4. PGPB and Its Role in Inducing Different Abiotic Stress Tolerance in Plants

**Figure 1:**
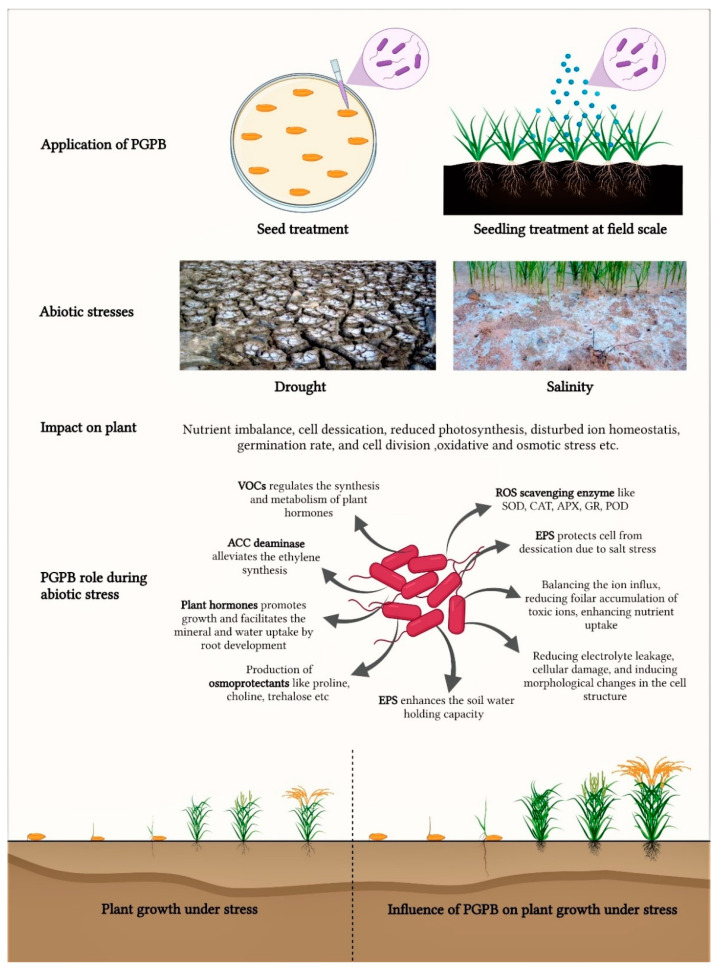
An overview of the role of PGPB in alleviating the effects of abiotic stress under salinity or drought.

Acclimatization of plants under drought and high salinity conditions is usually possible through natural physiological and biochemical adaptations [145]. Under conditions of prolonged or severe abiotic stress, the use of PGPR capable of tolerating these harsh conditions may be critial [53]. Inoculation of properly selected AST-PGPB can mitigate the effects of abiotic stresses by acting directly on plants or by positively modulating the plant’s natural defenses (Figure 1) [18,48,146].

### 4.1. PGPB Induced Salinity Tolerance

Selected salinity-tolerant bacteria are able to produce hormones, ACC deaminase, osmoprotectants or secondary compounds, such as EPS and VOCs, under high-salinity conditions (Figure 1) [55,119,123,130]. AST-PGPB inoculation may lead to positive adaptive responses of plants to salinity stress. This can occur through several mechanisms, including altered hormone production by the plant, increased nutrient uptake, lowering water stress, maintainance of favourable K^+^/Na^+^ ratio or osmotic adjustment (Table 1) [52,67,123]. Several genera are involved in the successful control of salinity stress in crops, such as *Bacillus*, *Pseudomonas*, *Agrobacterium*, *Streptomyces* or *Ochromobacter* [52,105,147,148]. Many studies have been conducted to verify the prevalence of salinity-tolerant strains. The dominance of 8% NaCl-tolerant *Bacillus* sp. in the wheat rhizosphere was observed [149]. Zhang et al. reported that of 305 bacteria isolated from paddy soil in Taoyuan, China, 35.7%, 15.1% and 4.9% of the strains were able to grow in media with 5%, 10% and 15% NaCl concentrations, respectively [150]. Most of these isolates exhibited plant growth promoting potential in rice cultivation under high salinity stress. Moreover, phylogenetic analysis of 74 selected isolates revealed that most of bacteria belonged to the order Bacillales. However, one of the best salinity tolerances for field strains, up to 20%, was found for *Klebsiella* sp. IG3 isolated from wheat rhizosphere [151]. In addition, it has been reported that multispecies inoculum consisting of endophytic and rhizosphere PGPB under salinity stress may increase crop yield [13].

Many studies have proven that selected bacterial strains are able to mitigate the overall negative effect under salt stress conditions. *Pseudomonas* sp. M30-35 isolated from *Haloxylon ammodendron* improved the growth and total biomass of quinoa under 300 mM NaCl [152], while *Azotobacter vinellandii* SRIAz3, isolated from the rice rhizosphere, increased fresh biomass under 200 mM NaCl and perennial ryegrass under 150 and 300 mM NaCl [24,153]. The mitigation of the effects of salt stress by AST-PGPB may occur at the level of gene transcription. The comparative transcriptome study showed a change in the activation of multiple genes in maize inoculated with *Bacillus amyloliquefaciens* SN13 [80]. In addition, 34 genes with homology to genes associated with different PGP attributes were described in salt-tolerant *Pseudomonas* sp. M30-35 [24].

The salinity tolerance of plants may result from their ability to maintain a high concentration of K^+^ to Na^+^ ions or to suppress the influence of Na^+^ ions on the root cells [52,154,155]. The natural mechanism of bacterial salinity tolerance is to avoid high salt concentrations in the cytoplasm. Salinity-tolerant bacteria can eliminate the excess salt by Na^+^/H^+^ anitiporters or prevent salt from flowing inside [13]. This can make the bacteria extremely resistant to high salinity, even if they occur naturally in a low salinity environment. Rice inoculated with *Bacillus pumilus* resulted in limited uptake of Na^+^ under 150 mM NaCl. The reduction of a toxic high level of Na^+^ ions resulted in an increase in shoot growth compared to the control [156]. Sunflower inoculated with *P. fluorescens* CECT 378^T^ under 100 mM NaCl salinity also increased the K^+^/Na^+^ ratio in the shoot, which supported the accumulation of plant biomass [157]. *P. putida* Rs-198 increased Mg^2+^, K^+^ andCa^2+^ and decreased Na^+^ uptake by soil affected cotton seedling roots [158]. *Rhizobium* and *Pseudomonas* lead to lack of Na^+^ accumulation in maize salinity affected [53]. In turn, maize inoculated with *Azotobacter chroococcum* enhanced Na^+^ exclusion and K^+^ uptake in maize, which also increased the K^+^/Na^+^ ratio. This experiment was performed under different salinity conditions, but plant promotion was observed only under 2.93 and 5.85 g NaCl/kg concentrations [159].

Some AST-PGPB may increase nutrients availability to plants by chelation or acidification of soil (Figure 1). Phosphorus, potassium, nitrogen or zinc deficiency in soil could be minimized through bacterial solubilization activity, thus dissolving both inorganic and organic phosphorus and to maintain soil nutrient levels [21,49,62]. Phosphate mobilization along with other PGPB traits, such as IAA and siderophore production, enhanced chlorophyll content in pepper inoculated with *M. oleivorans* KNUC7074, *B. iodinum* KNUC7183, or *R. massiliae* KNUC7586, under 200 mM NaCl [160]. Phosphate solubilization activity was also reported by Habib *et al*., where rice was inoculated *Bacillus* sp. UPMR7 and *Citrobacter* sp. UPMR17., and by Palaniyandi *et al*., where tomato was inoculated with *Streptomyces* sp. PGPA39 [143,161]. Likewise in these experiments, an increased amount of chlorophyll was observed. Phosphate solubilization also has been reported by Hahm *et al*., where *Rhizobium massiliae* KNUC7586, *Microbacterium oleivorans* KNUC7074 and *Brevibacterium iodinum* KNUC7183, through IAA and siderophore production, increased leaf water content in pepper, under 200 mM NaCl [160]. Phosphate solubilization is also crucial for root system improvement. Various strains, such as *Streptomyces* sp., *Bacillus* sp. and *Arthrobacter pascens*, used for wheat, rice and maize crop, respectively, resulted in root system growth enhancement [142,144,162]. Salt-tolerant phosphate solubilizing strains *B. pumilus* FAB10 increased wheat yield under salinity stress up to 250 mM NaCl [163]. Some phosphate solubilizers increased shoot length in pepper and rice under 200 mM and 150 mM NaCl, respectively [141,164]. Wheat inoculated with phosphate solubilizer *Streptomyces* spp. increased uptake of nitrogen, iron, phosphorous and manganese by the roots [165]. It has been demonstrated that maize inoculated with *Rhizobium* or *Pseudomonas* increased selective ion uptake under salinity conditions. Particular bacteria are able to dissolve other crucial minerals for plant growth. Ashfaq et al. have reported about halotolerant rhizobacteria, which may improve potassium availability under salinity stress [112]. Twenty-nine isolates out of 50 halophilic rhizobacteria were able to solubilize zinc carbonate and zinc oxide, under 3% NaCl concentration, while 10 isolates solubilized zinc carbonate under 10% concentration of NaCl [139]. It has also been reported that selected salt-tolerant PGPB may also increase iron, copper, zinc, manganese content due to their activity [13,125,166,167]. 

Regulation of cell membrane permeability and thus control of water movement through organs is an important adaptive mechanism under osmotic stress, caused by high salinity [10,72]. The accumulation of proteins or amino acids in leaves helps protect cell structures from denaturation in the event of dehydration. Certain osmolytes can lower the osmotic potential under water stress. This osmotic adjustment helps maintain plant cell turgor under salinity and drought stresses [142,146]. It has been reported that maize co-inoculated with *Rhizobium* and *Pseudomonas* lowered osmotic potential due to enhanced proline production, resulting in higher water content in leaves [53]. Additionally, peanut inoculated *Brachybacterium saurashtrense* (JG-06), *Brevibacterium casei* (JG-08) or *Haererohalobacter* (JG-11) increased shoot and root water content due to higher proline and soluble sugar production [53]. Chickpea inoculated with *A. lipoferum* FK1, also showed higher accumulation of proline and glycine-betaine, as well as proteins and sugars under salinity stress exhibited [168]. Rice inoculated with *B. amyloliquefaciens* SN13 and *Azotobacter vinellandii* SRIAz3 produced more proline, resulting in increased plant biomass [80,169].

AST-PGPB could directly affect plant metabolism through hormone production, thus enhancing plant growth under saline conditions [7,52,170]. In addition, some of the AST-PGPB hormone producers may also shift the synthesis of selected hormones in plants under various abiotic stresses [157,158]. It has been reported that IAA producing bacteria are very common within the AST-PGPB group [24,83,171]. Pepper inoculated with IAA-producing bacteria *M. oleivorans* KNUC7074, *B. iodinum* KNUC7183, and *R. massiliae* KNUC7586 increased chlorophyll concentration, as well as leaf water content under 200 mM NaCl salinity [160]. A similar effect was reported in rice inoculated with *Bacillus* sp. UPMR7 and *Citrobacter* sp. UPMR17 or tomato crop using *Streptomyces* sp. PGA39 [172,173]. Yao et al. studied the growth promotion of cotton seedling inoculated with *P. putida* Rs-198 [158]. Germination rate increased 23.8% under saline soil conditions compared to control conditions. Moreover, cotton seedlings inoculated with strain *P. putida* Rs-198 improved production of IAA and reduced ABA synthesis, which resulted in increased Mg2+, K+ and Ca2+ and decreased Na+ uptake by roots [158].

It has been widely reported that IAA producers among AST-PGPB play an important role in improving root system architecture and length [57,64]. IAA promotes root initiation, reinforcement of lateral and adventitious roots, which increases water absorption surface. Sadeghi et al. reported that *Streptomyces* sp. increases N, Fe, P, and Mn uptake by wheat due to IAA and siderophore production and phosphate solubilization [165]. Yasin et al. observed increased root length of pepper by 87.3% and 69.8% in salt-stressed conditions using *Bacillus fortis* SSB21 and SSB13 strains, respectively, compared to control [174]. The experiment also proved that auxins play a special role in promoting plant growth, especially lengthening the shoot. Pepper-inoculated IAA producers *M. oleivorans* KNUC7074, *R. massiliae* KNUC7586 and *B. iodinum* KNU7183 increased shoot length by 33%, 35% and 37%, respectively, compared to control, under 200 mM NaCl [160]. Further, there are cytokinin, gibberellin and ABA PGPB producers [125]. It has been reported that *Pseudomonas putida* and *Novosphingobium* sp. are able to decrease the production of the hormones ABA and SA in citrus plants under salinity stress. An increase in these hormones was observed in control [175]. 

Ethylene as a gaseous hormone regulates growth, senescence, and stress tolerance in plants at low concentrations [55,149], while increased concentration of ethylene negatively affects root growth. Plants produce this gas from 1-aminocyclopropane-1-decarboxylate (ACC). The enzyme ACC deaminase degrades ACC to α-ketoglutarate and NH_3_, thus preventing further ethylene production. ACC deaminase-producing microbes such as actinomycetes can uptake ACC, metabolize it, and thus lower the ethylene concentration in the plant [55,98,176]. Some bacterial species, such as *Pseudomonas* sp., *Variovorax paradoxus* and *Rhizobium phaseoli*, are great salinity alleviators due to ACC deaminase activity [114,170,177]. Selected ACC deaminase producers at AST-PGPB may show other properties. *Streptomyces* sp. PGPA39-inoculated tomato under salinity stress was able to alleviate salinity stress by phosphate solubilization, ACC deaminase and IAA production [173]. This strain enhanced chlorophyll and water content. Rice inoculated with ACC-deaminase-producer *Curtobacterium albidum* SRV4 increased yield under salt stress, compared to controlled conditions [178]. Wheat inoculated with *B. pumilus* FAB10 increased yield by 10.2% under 250 mM NaCl concentration [163]. Moreover, *Burkholderia* sp. MTCC was able to promote the growth of rice seedlings due to ACC deaminase production and higher antioxidant activity [54].

Some of the AST-PGPB can enhance photosynthesis under salinity stress. Ribulose-1,5-bisphosphate carboxylase/oxygenase (EC 4.1.1.39), commonly known as RuBisCo, plays crucial role in CO_2_ fixation during the dark stage of photosynthesis [179]. Positive up-regulation of rbcL, gene encoding RuBisCo in the oat seedlings inoculated with *Klebsiella* sp. under 100 mM NaCl salinity stress, resulted in enhanced photosynthetic activity [151]. Moreover, photosynthesis may be intensified as a result of the increased chlorophyll content. In addition, inoculation of bacteria, which exhibit typical growth promotion traits such as IAA production, phosphate solubilization or N-fixation were able to increase chlorophyll content in pepper, rice or tomato crops [160,172,173]. 

As mentioned earlier, EPS makes bacteria more tolerant to osmotic stress. EPS can participate in the control of stomatal movement, hydraulic conductance and transpiration, and maintains integrity of plant cells [67]. These actions allow the plant to maintain adequate hydration. The positive effect of EPS on proper hydration through improved K^+^/Na^+^ ratio was demonstrated by Yang et al. in which quinoa was inoculated with *Enterobacter* sp. MN17 and *Bacillus* sp. MN54 under 100 mM NaCl [58]. Decreased Na^+^ uptake reduces osmotic stress, thus producing enhanced plant growth. Production of EPS by *Rhizobium* sp. IC3123 under 16 mM NaCl enhanced the germination process, seed yield and nodule formation in the pigeon pea, in both field and pot experiments [161]. Tewari and Arora reported the influence of EPS producer *P. aeruginosa* P23 on increased sunflower yield under 500 mM NaCl [142]. In addition, it was revealed that EPS can enhance plant hormone production. Sunflower inoculated with *P. aeruginosa* P23 under 500 mM NaCl, and canola inoculated with *B. iodinum* RS16, *Micrococcus yunnanensis* RS222, *Brevibacterium aryabhattai* RS341 and *Bacillus licheniformis* RS656, under 100 mM NaCl, resulted in higher production of SA and growth hormones, respectively [165,173]. Moreover, its properties could result from its ability to form a biofilm on plant roots, especially under high salinity. *B. pumilus* FAB10 showed biofilm formation on the surface of wheat roots at a salt concentration of 250 mM. *B. pumilus* increased biofilm synthesis with increasing salinity, between 125 and 250 mM NaCl, which was accompanied with higher EPS production [141].

Selected AST-PGPB are able to produce high amount of antioxidant enzymes, mainly SOD, POD, CAT, GR and nitrate reductase (NR), under salinity stress [78,174,180,181]. Reduced production of antioxidant enzymes is observed in situations with low tolerance to salt stress in the plant. In such cases, it is worth supporting their growth with bacteria that can produce such enzymes in high concentrations [53]. Plants inoculated with selected bacteria under salinity stress conditions can increase the synthesis of these enzymes by the plant itself. Their action may simply assist plants to synthesize such enzymes. This creates an opportunity to use bacteria with increased salinity tolerance to mitigate the effects of salinity stress, even if they do not produce these enzymes. Chickpea inoculated with *A. lipoferum* FK1 resulted in increased production of antioxidant enzymes. This strain enhanced the transcription of three antioxidant genes encoding CAT, APX, and SOD, as well as six other genes involved in the alleviation of abiotic stress [168]. The enhanced antioxidant enzymes’ production stimulated the growth of chickpeas due to the increased protection of chloroplasts. Kohler et al. have been reported that *Pseudomonas mendocina* increased APX and CAT production in lettuce under salinity conditions [182]. Inoculation of these bacteria reduced the damage from oxidative stress and increased the shoot biomass. Wheat inoculated with *Stenotrophomonas maltophilia* SBP-9 isolated from *Sorghum bicolor*, resulted in increased production of SOD (26–41%), CAT (24–56%), and POX (27–44%) [183]. Wheat seeds inoculated with *B. pumilus* FAB10 resulted in decreased salinity stress, which was manifested by a decrease in the production of selected antioxidant enzymes [141]. Wheat seeds treated with *B. pumilus* FAB10 showed a 20% increase in CAT production despite different salinity concentrations: 75, 125, and 250 mM NaCl, compared with control conditions. While unprimed seeds increased of CAT enzyme production, relative to control 40%, 80% and 80% for 75, 125, and 250 mM NaCl, respectively. A significant decrease in SOD and GR production was also observed as compared to seeds untreated with *B. pumilus* FAB10. Has been reported that rice seeds treated with halotolerant *Enterobacter* P23 under 150 mM NaCl decreased level of SOD, CAT and peroxidase in plants [184]. These data may indicate that appropriate strain adjustment under specific conditions can bring great benefits despite harsh environmental conditions. Due to the high production of ROS by plants under salinity stress, the quenchers of these molecules are critical for crop survival. Rice inoculated with *Pseudomonas pseudoalcaligenes* and *B. pumilus* reduced lipid peroxidation process and SOD under 1.5% saline conditions [53]. Rice inoculated with *A. vinellandia* SRIAz3, isolated from the rice rhizosphere, shows a high concentration of proline and MDA under 200 mM NaCl [169].

Although salinity can seriously affect crop growth and yield, there are numerous salinity-tolerant microorganisms are available, as seen above. Due to their several beneficial traits, they are able to mitigate the effects of stress in various crops. However, it should be kept in mind that the effect of inoculation on crop health and yield depends on a number of factors besides soil and nutrient status. Therefore, it is necessary to constantly study the influence of microorganisms with different characteristics on counteracting the effects of salt stress.

### 4.2. PGPB Induced Drought Tolerance

Soil water shortage adversely affects plants, often inhibiting their proper growth and development. Less access to water increases the concentration of ions. Both water scarcity and high salinity can create hypertonic conditions, leading to osmotic stress, thus disturbing the nutrient and water balance, the permeability of membranes and and reducing the activity of selected enzymes [147]. Drought tolerance is usually the result of many biochemical and physiological adaptations, which consequently allow the plant to maintain its desired size and yield despite unfavorable environmental conditions. However, in the case of long-term or sudden changes, the plant is unable to cope and needs external help to survive.

Abiotic stress-tolerant PGPB may beneficially influence the plant growth and development under water deficit conditions, thus conferring tolerance to drought stress (Figure 1). There are several mechanisms of AST-PGPB that lead to increased water absorption despite drought symptoms (Table 1) [185]. AST-PGPB may confer drought tolerance in plants mainly by (i) hormone production, (ii) ACC deaminase production, (iii) osmoprotectant accumulation, (iv) solubilization activity and (v) regulation of stress-responsive genes (Figure 1) [6,111,186]. There are several genera that are used to alleviate drought stress in crops, such as *Azospirillum*, *Bacillus*, *Pseudomonas* or *Microbacterium* [135,137,163,186]. Most of drought tolerant PGPB strains are plant hormone producers. In lower concentrations, auxin, as mentioned before in this paper, promotes root growth and branching and supports overall vigorous root growth, and thus water and nutrient uptake capacity [47,186,187]. Increased ABA, GA and IAA reduced drought stress due to maize inoculated endophytic *A. lipoferum.*

Bacterial GA and ABA increased leaves compared to the control treated with these hormone inhibitors under drought stress [188]. The experiment proved a significant role of these hormones in plant growth and drought tolerance. In addition, JA plays a role in alleviating drought stress by increasing antioxidant activity. Therefore, bacteria increasing JA hormone production may enhance drought tolerance [189]. *P. putida* H-2-3 mitigated the drought and salinity stress effects on soybean by GA production ability. Soybean inoculated *P. putida* H-2-3, compared to the control under drought conditions, exhibited slightly better chlorophyll content (1.2%), but primarily a greater shoot length (13.6%) and plant fresh weight (12.8%) [13]. Sarma and Saikia have reported that mungbeans inoculated with *Pseudomonas aeruginosa* GGRJ21 under 0.73 MPa drought conditions enhanced their biomass and growth in field conditions compared to control [109]. Bacterial inoculation promoted root length in the plants by 127% and shoot length by 42% under water stress conditions. The elongation of the roots and shoots was possible due to IAA production and the stress reduction effects through the ACC deaminase activity. Moreover, bacterial up-regulated transcription of stress responsive genes, which contributes to the plant stress tolerance [109]. Cytokinin as a natural plant hormone supports the young plant, thus preventing leaves scarcity. However, its synthesis during drought is limited [189]. Inoculation of cytokinin-producing strain *B. subtilis* on *Platycladus orientalis* elevated cytokinin in shoots under drought stress [190]. It was noted that GA plays a crucial role in the control of the degree of opening of the stomata, and therefore is responsible for the process of stomatal transpiration. In times of drought, access to water is negligible, so closing the apparatuses may reduce water losses [191]. Bacterial inoculation elevated ABA and reduced drought stress. Cucumber inoculated with of *P. putida* enhanced shoot length and biomass due to higher endogenous GA production in plants [13]. *Azosprillum brasilense* Sp245 inoculation elevated ABA and reduced drought stress in *Arabidopsis* [192]. *Phyllobacterium brassicacearum* STM196 inoculated with rapeseed increased osmotic stress tolerance under drought stress due to elevated ABA concentration. Moreover, *A. brasilense* due to nitric oxide production enhanced adventitious root development in tomato, which could be another mechanism of drought stress alleviation [193]. 

Maize inoculated with *A. brasilense* enhanced proline accumulation, plant water content, biomass and leaves area of plant under drought stress [192]. Additionally, it has been reported that wheat inoculated with *A. brasilense* Sp245 under drought conditions increased yield, mineral uptake and water content in the plant [194]. Similarly, maize inoculated with *P. putida* GAP-45 increased relative water content due to proline accumulation under drought stress [195]. Increased proline accumulation was also observed in *Lavandula dentata* inoculated with *B. thuringiensis* and tomato inoculated with *Bacillus polymyxa* [196,197].

Intact and proper functioning of the plant requires undisturbed photosynthesis, which is a key process that provides energy compounds and substrates for plant development. Maintaining the optimal turgor of leaf cells is crucial in the proper performance of photosynthesis, resulting in a high photosynthetic rate. Regulating the opening of stomatal apertures ensures proper hydration of the plant, as well as water uptake from the soil [124]. ABA hormone may regulate the closure of stomata under drought to avoid water loss [42]. Some AST-PGPB also have the ability to produce osmoprotectants under abiotic stress, particularly drought [105]. The accumulation of these compounds decreases the water potential in the leaves and thus their natural transport from stems by suction force. Plants treated with PGPB may exhibit higher content of antioxidant enzymes and cell osmolytes [109]. Wheat inoculated with *Azospirillum* sp. maintained hydration as a result of decreased leaf water potential and increased leaf water relative content due to auxin production, phosphate solubilization and ACC deaminase activity by bacteria [63]. 

Improvement of leaf water status in mung bean under drought conditions was reported [109]. *Pseudomonas aeruginosa* GGRJ21 resulted in proline synthesis and enhanced accumulation along with antioxidant enzymes in plants. Trehalose, natural non-reducing sugar accumulation, has been found to be osmoprotective in both bacteria and plants. It may also be involved in enzymes and membrane stabilization [192]. The experiments conducted by Vílchez et al. using a collection of desiccation-tolerant microorganisms revealed again the role of trehalose, as well as hormones produced by bacteria [111]. Pepper and tomato treated with *Microbacterium* sp. 3J1 under severe drought conditions showed the highest values of relative water content, dry weight and fresh weight. This strain showed the highest production of ACC deaminase and was able to synthesize the hormones IAA, GA and SA. Trehalose can regulate the expression of genes responsible for the production of antioxidant enzymes, as well as modulate their activity [111]. Moreover, modification of the *P. putida* KT2440 strain to overproduce trehalose resulted in increased plant tolerance to desiccation [111]. Other osmoprotectants’ accumulation, such as glycine betaine, may increase water content. Maize inoculated with *Klebsiella variicola* F2, *P. fluorescens* YX2, and *Raoultella planticola* YL2 and *Arabidopsis* inoculated *B. subtilis* GB03 due to choline accumulation, as a precursor of glycine-betaine increased water content, thus enhancing drought tolerance of this plant [198,199]. Drought, similar to high salinity conditions, may generate a large number of ROS molecules in plants. Reducing the negative effects of stress, especially biomass losses caused by the formation of a high concentration of ROS, is possible thanks to scavenging mechanisms [65,112]. Lower content of antioxidant enzymes compared to control conditions may indicate drought stress amelioration. For instance, rice inoculated with *Bacillus* sp., as well as with a particular PGPB consortium, resulted in decreased levels of GPOX (20%), CAT (20.5%) and SOD (24%) in plants compared to those untreated, under drought stress [200].

Water scarcity decreases the rate of photosynthetic CO_2_ assimilation due to stomatal closure. Severe dehydration induces photorespiration as a result of both low CO_2_ concentration and the unavailability of a sufficient amount of water. Particular antioxidative enzymes, such as CAT and APX, seem to play an important role in plant tolerance to drought stress [205]. Prolonged drought can interfere with photosynthesis due to the reduced amount of chlorophyll, which in turn reduces plant growth and yield. 

Slower root growth under drought stress conditions reduces the area of uptake of both water and nutrients. Wheat inoculated with three *Azospirillum* sp. strains AZ1, AZ9 and AZ45 increased yield compared to uninoculated treatments under varied drought stress. AZ45 increased wheat yield by 43% and 109% due to N fixation, IAA, ACC deaminase production and phosphate solubilization [63]. Relative water content was increased by this strain by 6.1% and 1.8%, while strain AZ1 raised relative water content by 3.3% and 7.8% due to N fixation, IAA and ACC deaminase production. In addition, maize inoculated with *Bacillus* sp. increased K^+^ and P^+^ uptake, as well as proline accumulation under drought stress, which resulted in increased leaf biomass [88]. Increased phosphates uptake occurred due to the phosphorous solubilization properties of strains. The authors mentioned the correlation between proline accumulation and root growth, the activity of antioxidant enzymes and the uptake of K^+^ ions. Proline itself exhibits an antioxidant capacity and is involved in the stabilization of membranes and proteins, and thus participates in osmotic adjustment, which seems to be crucial in drought conditions [88]. Water stress may also decrease the nitrate reductase activity in plants and bacteria, thus leading to nitrate accumulation and inhibiting plant growth. However, due to the hormones produced by plants inoculated by *Azospirillum* strains, increased nitrogen and other nutrient uptake was observed [63,211]. Drought also affects the composition of plant cell membranes. It has been reported that *Azospirillum* strains can also prevent negative changes in membrane composition in wheat seedlings [192]. 

Low concentrations of ethylene have a positive effect on adventitious root development and fruit ripening, while high production of ethylene under abiotic stress conditions, including drought, leads to many harmful effects, such as inhibition of root and shoot growth, defoliation and premature senescence [6,114]. Saikia et al. reported on the potential benefits of employing a consortium of ACC-deaminase generating bacteria to alleviate drought stress in black gram and garden pea [105]. Other plants, such as tomato and pepper, inoculated with ACC deaminase producing *Achromobacter piechaudii* ARV8, showed beneficial impacts, such as increased growth, particularly fresh and dry weights, under drought stress [202]. Garden pea inoculated with the ACC deaminase producer *P. fluorescens* resulted in longer roots, and thus increased water uptake, under drought stress [207]. 

Plants exposed to drought stress may produce VOCs as a signal molecule involved in plant tolerant responses. Alleviation mechanisms could be based on properties of VOCs produced by AST-PGPB or enhancing synthesis of these molecules in plants due to bacteria’s other activities [192]. It has also been reported that 2R, 3R- butanediol released by *P. chlororaphis* O6 reduces water loss by *Arabidopsis* due to better control of stomata closure under drought conditions [205]. Another mechanism to counteract drying out is the EPS accumulation produced by AST-PGPB. Bacteria due to EPS production can produce a biofilm, which creates a microenvironment beneficial for both sides, bacteria and plant, for a long time [168,189]. EPS-coated roots are well protected from desiccation, making them less exposed to the negative effects of drought stress. In addition, bacterial EPS released into the soil can improve soil structure, retain soil water, and increase bacterial colonization on the roots [215]. EPS’s special role in drought stress alleviation has been reported by Sandhya et al. [195]. Sunflower inoculated with *P. putida* GAP-45 increased relative water content. Moreover, these bacteria enhanced soil aggregation. EPS production has a positive influence on osmolytes accumulation, thus enabling water adjustment in plants. Under the influence of EPSproducing bacteria, various selected plants have been observed exhibiting reduced levels of antioxidant enzymes [118,120,159]. 

In conclusion, drought tolerance in crops can be increased by inoculation with drought-resistant bacteria. When selecting an appropriate microorganism, the intensity of drought, its length, other climatic factors and the type of crop should be taken into account. Among the bacteria, there are species that cope well with the conditions of a low level or lack of water in soil. Strains belonging to *Azospirillum*, *Pseudomonas* and *Bacillus* are among the most promising potential candidates for AST-PGPB due to their extreme drought tolerance and display numerous PGPB characteristics. EPS-producing bacteria cope best in drought conditions, while plant growth and reduction of drought stress is possible thanks to ACC deaminase activity and hormone production.

### 4.3. Challenges and Limitations Associated with PGPB Application

There are a number of articles, some of which have also been discussed in this article, illustrating the benefits of PGPB in boosting plant growth and development under abiotic stress [5,54,67,216]. The beneficial role of PGPB has already been confirmed by successful experiments in laboratories and under controlled conditions. Despite having a fair understanding of microbial efficiency, there is still a need for rigorous research to translate these approaches to the field [67,113,123,155]. To bridge this significant gap in the use of microbes or microbe-based chemicals from laboratory to field scale, it is necessary to comprehend the various issues related to inconsistencies, ambiguities and problems in application methods, etc. According to several researchers, one of the reasons for the poor performance of the microbial community under natural circumstances is a lack of information about the optimal carrier in traditional formulations (solid and liquid) [4,171,217]. The carrier material used to make a suitable formulation is critical for protecting the microorganisms from adverse conditions throughout storage, transportation, and their residence in the soil [217,218]. The microbial formulations are available primarily in two forms: liquid suspensions and solid powders or beads. Peat, turf, lignite, alginate, press mud, vermiculate and zeolite are some of the carrier materials utilized in formulation. These carriers may provide nourishment to the microbial community. On the other hand, they can protect the microbes from harsh environments and desiccation, thus extending their shelf life. The marketing and commercialization of microbial-based products depends on market demand, consistency, broad spectrum action, environmental safety, cheap capital cost, environmentally friendly behavior and strong collaboration between industry and research [120,219,220]. Some of the major factors limiting the success of PGPB are: (i) crop specificity of PGPB products; (ii) selection of potent PGPB isolates for product formation; (iii) farmers’ attitude; (iv) stability of PGPB formulation; (v) performance under different climatic conditions; and (vi) lack of skilled manpower for microbial product application.

## 5. Conclusions and Future Prospects

The continuous increase in energy consumption together with land use for agriculture leads to the GHG emissions that trap solar radiation in the atmosphere. Variation in precipitation, melting glaciers, reduced snow cover and rising ocean levels are the main signs of climate change caused by an increase in average temperature. It is likely that as climate change progresses in the 21st century, significant areas of high-quality farmland will be lost to due to sea rise, erosion, salinization and desertification [13,20]. Climate change is constantly exacerbating the problem of declining available cropland and freshwater resources. This aggravates the imbalance between wealthy and developing human communities. Underdeveloped nations, particularly on the African continent, are expected to be the hardest hit by elevated temperatures [53]. Climate change will only worsen the existing problems of water scarcity and the prevailing drought. Projections indicate that some crops will no longer be possible due to water shortage or soil degradation. On the other hand, more developed countries, especially southern and eastern Australia and New Zealand, will see their crop significantly reduced due to rising temperatures and drought. In particular, the struggle with drought and salinity is one of the toughest challenges in agriculture since many decades ago [217].

One of the greatest challenges facing mankind in the times ahead is to provide for the possibility of cultivation despite growing problems related mainly to drought and salinity. It is important that rigorous regulations and particular techniques for the application of efficient biofertilizers are developed in order to sustainably control abiotic stressors such as drought and salt. Several investigations have established their effectiveness in improving the growth and yield of crops under salinity and drought stress. Using natural resources while stopping further degradation is one of the ways to meet the food requirements of a growing society in a changing climate. Sustainable agriculture should cooperate with the natural environment, using only solutions compatible with it, in order to be able to mitigate the effects of a changing climate without risking even greater degradation [14]. Adaptation and mitigation to climate change are pose a challenge in agriculture nowadays and in the near future. The use of stress-resistant PGPB may not only help in the fight against the effects of abiotic stresses, but also lead to the mitigation of climate change, as a result of discontinuing the use of some non-environmentally friendly solutions, such as chemical fertilizer usage. The short storage and shelf-life of microbe-based formulations is a significant constraint. The carrier material used to create a proper formulation is essential in preserving microorganisms from harmful conditions during storage and transit, as well as extending their shelf life. One of the causes for the microbial community’s poor performance in natural conditions is a lack of knowledge about the suitable carrier in conventional formulations (solid and liquid). According to some research, microencapsulation is a cutting-edge technique that may be utilized successfully to address these disadvantages. In the future, more rigorous studies are required to evaluate their additive effect on plant growth, and to study, at the molecular level, plant-microbe interaction during multiple abiotic stressors under field conditions and to study the different carrier materials for appropriate microbial formulation with longer shelf lives.

## Figures and Tables

**Table 1:** Plant growth-promoting bacteria (PGPB) and their induced changes in plant morphological and physiological molecular traits with alleviation mechanisms under drought and salinity stresses.

Abiotic Stress	Effect on Plant	Alleviation Mechanism	PGPB	Crop	References
Drought	Increased plant growth	GA production by bacteria. Decreased level of ABA and salicylic acid, higher level of JA in plants.	*Pseudomonas putida* H-2-3	Soybean (*Glycine max* L.)	[201]
	Increased plant biomass	IAA, GA, SA production, ACC activity by bacteria.	*Microbacterium* sp. 3J1	Tomato (*Solanum Lycopersicum* L.)	[111]
				Pepper (*Capsicum annum* L.)	
		Enhanced proline synthesis by plants.	*Azospirillum brasilense*	Maize (*Zea mays* L.)	[192]
		Increased K+ and P+ uptake, as well as proline accumulation in plants.	*Bacillus* sp.	Maize (*Zea mays* L.)	[88]
		Decreased level of GPOX, CAT and SOD in plants.	*Bacillus* sp.	Rice (*Oryza sativa* L.)	[200]
		ACC deaminase production by bacteria.	*Achromobacter piechaudii* ARV8	Tomato (*Solanum Lycopersicum* L.)	[202]
				Pepper (*Capsicum annum* L.)	
		Upregulation of stress protein genes *Cadhn*, *VA*, *sHSP* and *CaPR-10*	*B. licheniformis* K11 →	Pepper (*Capsicum annum* L.)	[203]
	Leaf water status improved	Phosphate solubilization, ACC deaminase, IAA, HCN and siderophores production by bacteria. Enhanced accumulation of antioxidant enzymes and proline in plants.	*P. aeruginosa* GGRJ21	Mung bean (*Vigna radiata* L.)	[109]
		Auxin production by bacteria.	*Azospirillum lipoferum* AZ45	Wheat (*Triticum aestivum* L.)	[63]
		Enhanced proline synthesis by plant.	*A. brasilense*	Maize (*Zea mays* L.)	[192]
		Enhanced proline synthesis by plant. Increased cytokinin level in shoots and root exudates in plants.	*P. putida* GAP-45	Maize (*Zea mays* L.)	[195]
			*B. subtilis*	*Platycladus orientalis*	[190]
		IAA production, P solubilization, ACC deaminase activity by bacteria.	*Azospirillum* sp.	Wheat (*Triticum aestivum* L.)	[63]
		Elevated ABA concentration in plants.	*A. brasilense*	*Arabidopsis thaliana* L.	[192,204]
	Reduced water loss	2R, 3R- butanediol released by bacteria.	*P. chlororaphis O6*	*Arabidopsis thaliana* L.	[205]
		Choline accumulation, as a precursor of glycine betaine in plants.	*Klebsiella variicola* F2, *P. fluorescens* YX2 *and Raoultella planticola* YL2	Maize (*Zea mays* L.)	[198]
	Increased water content	Choline accumulation, as a precursor of glycine betaine in plants.N fixation, IAA and ACC deaminase production by bacteria.	*B. subtilis* GB03	*Arabidopsis thaliana L.*	[199]
			*Azospirillum* sp. AZ1	Garden pea (*Pisum sativum* L.)	[63]
		EPS production by bacteria.	*P. putida* GAP-45	Sunflower (*Helianthus annuus* L.)	[195]
		Upregulation of stress specific genes *APX1*, *SAMS1*, and *HSP17.8*	*Bacillus amyloliquefaciens* 5113 and *Azospirillum brasilense* NO40	Wheat (*Triticum aestivum* L.)	[206]
		IAA-production by bacteria.	*Klebsiella* sp. IG 3	Wheat (*Triticum aestivum* L.)	[20]
	Enhanced length and number of roots	ACC deaminase and IAA production by bacteria.	*P. aeruginosa* GGRJ21	Mung bean (*Vigna radiata* L.)	[109]
		ACC deaminase production by bacteria.	*P. fluorescens*	Garden pea (*Pisum sativum* L.)	[207]
	Longer roots	Nitric oxide production by bacteria.	*A. brasilense*	Tomato (*Solanum Lycopersicum* L.)	[193]
	Enhanced adventitious root development	Increase of IAA content in plants.	*B. subtilis* LDR2	Wheat (*Triticum aestivum* L.)	[20]
	Higher photosynthetic efficiency	N fixation; auxin and ACC deaminase production; P solubilization by bacteria.	*A. lipoferum* AZ45	Wheat (*Triticum aestivum* L.)	[63]
	Higher growth and yield	Decreased level of GPOX, CAT and SOD in plants.	*Bacillus* sp.	Rice (*Oryza sativa* L.)	[200]
		N fixation, IAA and ACC deaminase production, P solubilization by bacteria.	*Azospirillum* sp. AZ45	Wheat (*Triticum aestivum* L.)	[63]
		ABA-dependent signaling genes activation	*Gluconacetobacter diazotrophicus* PAL5	Sugarcane (*Saccharum* spp.)	[208]
		ACC deaminase production by microbial consortium Downregulation of ACC-oxidase gene expression.	*Ochrobactrum pseudogrignonense* RJ12, * Pseudomonas s*p. RJ15 *and B. subtilis RJ46*	Black gram (*Vigna mungo* L.), Garden pea (*Pisum sativum* L.)	[109]
	Increased chlorophyll synthesis in leaf	Gibberellin production by bacteria.	*P. putida* H-2-3	Soybean (*Glycine max* L.)	[201]
		Up-regulation of expression profile of *rbcL* gene and down-regulation of *WRKY1* gene in plant.	*Klebsiella* sp.	Common oat (*Avena sativa* L.)	[151]
Salinity	Enhanced photosynthetic activity	IAA, siderophore production and phosphate solubilization by bacteria.	*Microbacterium oleivorans* KNUC7074, *Brevibacterium iodinum* KNUC7183, *Rhizobium massiliae* KNUC7586	Pepper (*Capsicum annum* L.)	[160]
	Enhanced chlorophyll content	IAA production, N–fixation, phosphate solubilization by bacteria.	*Bacillus* sp. UPMR7 and *Citrobacter* sp. UPMR17	Rice (*Oryza sativa* L.)	[172]
		Upregulation of Toc GTPase genes	*Pseudomonas putida* UW4	Tomato (*Solanum lycopersicum*)	[209]
		IAA production, phosphate solubilization ACC deaminase activity by bacteria.	*Streptomyces* sp. PGPA39	Tomato (*Solanum Lycopersicum* L.)	[173]
		IAA and siderophore production, phosphate solubilization by bacteria.	*M. oleivorans* KNUC7074, *B. iodinum* KNUC7183, *R. massiliae* KNUC7586	Pepper (*Capsicum annum* L.)	[160]
	Increased leaf water content	Enhanced proline synthesis by plant with reduction in electrolyte leakage, increased K^+^ uptake and decreased Na^+^/K^+^ ratio.	*Rhizobium* sp., *Pseudomona*s sp.	Maize (*Zea mays* L.)	[210]
		Increased proline content and soluble sugars, lower MDA in plants.	*Brachybacterium saurashtrense* (JG-06), *Brevibacterium casei* (JG-08), *Haererohalobacter* (JG-11)	Peanut (*Arachis hypogaea*)	[90]
	Increased shoot and root water content	EPS production by bacteria.	*Enterobacter* sp. MN17 and *Bacillus* sp. MN54	Quinoa (*Chenopodium quinoa* L.)	[58]
		Improved production of IAA and decreased ABA synthesis in plants;increased Mg2^+^, K^+^,Ca2^+^ and decreased Na^+^ uptake by roots.	*P. putida* Rs-198	Cotton (*Gossypium hirsutum* L.)	[158]
	Promoted seedling growth	ACC deaminase production, higher antioxidant enzymatic activities and decreased ethylene production in plants.	*Burkholderia* sp. MTCC 12,259	Rice (*Oryza sativa* L.)	[54]
		Lower level of ABA and SA in plants.	*P. putida* KT2440 or *Novosphingobium* sp. HR1a	Citrus plants	[175]
	Salinity damage prevention	Higher activity of peroxidase, catalase and nitrate reductase in plants.	*Pseudomonas* sp. PF1, *Pseudomonas* sp. TDK1	Rice (*Oryza sativa* L.)	[211]
		Upregulation of stress specific genes *RBCS*, *RBCL*, *H^+^-PPase*, *HKT1*, *NHX1*, *NHX2* and *NHX3*, as well as downregulation of *NCED* gene expression.	*Bacillus amyloliquefaciens* SQR9	Maize (*Zea mays* L.)	[212]
		Upregulation of ABA-signaling cascade genes *TaABARE* and *TaOPR1*; Enhanced expression of stress-induced gene *TaST*, *SOS1* and *SOS4.*	*Dietzia natronolimnaea* STR1	Wheat (*Triticum aestivum* L.)	[213]
		Higher accumulation of proteins, sugars, proline and glycine betaine in plants.	*A. lipoferum* FK1	Chickpea (*Cicer arietinum* L.)	[168]
		IAA production, phosphate solubilization, siderophore production, ACC activity in bacteria.	*Bacillus fortis*	Pepper (*Capsicum annum* L.)	[174]
	Improved root system	IAA production, N–fixation, phosphate solubilization by bacteria.	*Bacillus* sp. UPMR7 and *Citrobacter* sp. UPMR17	Rice (*Oryza sativa* L.)	[172]
		P-solubilization, bacteriocin and siderophore production by bacteria.	*Bacillus* sp., *A. pascens*	Maize (*Zea mays* L.)	[214]
		Increased proline content and totalsoluble sugar, decreased lipid peroxidation and electrolyte leakage in plants.	*B. amyloliquefaciens* SN13	Rice (*Oryza sativa* L.)	[80]
	Increased plant biomass	Increased IAA, GA, zeatin production; high proline and MDA content in plants.	*Azotobacter vinellandii* SRIAz3	Rice (*Oryza sativa* L.)	[169]
		ACC deaminase production by bacteria.	*Hartmannibacter diazotrophicus* E19^T^	Summer barley (*Hordeum vulgare* L.)	[204]
		IAA production, phosphate solubilization ACC deaminase activity by bacteria.	*Streptomyces* sp. PGPA39	Tomato (*Solanum Lycopersicum* L.)	[173]
		IAA and siderophores production, higher K^+^/Na^+^ ratio in shoot in plant.	*P. fluorescens* CECT 378^T^	Sunflower (*Helianthus annuus* L.)	[157]
		EPS production by bacteria. Growth hormones production by plant.	*Brevibacterium iodinum* RS16, *Micrococcus yunnanensis* RS222, *B. aryabhattai* RS341 and *B. licheniformis* RS656	Canola (*Brassica napus* L.)	[161]
		IAA production, N_2_ fixation, ACC deaminase activity, HCN and EPS production by bacteria.	*Curtobacterium albidum* SRV4	Rice (*Oryza sativa* L.)	[178]
	Increased yield	Biofilm producing on roots, enhanced amount of EPS, IAA production, ACC-deaminase activity, phosphate solubilization by bacteria.	*B. pumilus* FAB10	Wheat (*Triticum aestivum* L.)	[141]
		IAA production, N_2_ fixation, ACC deaminase activity, phosphate solubilization by bacteria.	*Azospirillum* sp.	Wheat (*Triticum*	[63]
		EPS production by bacteria.	*P. aeruginosa* P23	Sunflower (*Helianthus annuus* L.)	[161]
		IAA production, phosphate solubilization, siderophore production and ACC activity by bacteria.	*B. fortis*	Pepper (*Capsicum annum* L.)	[174]
	Increased shoot length	IAA production and phosphate solubilization by bacteria.	*M. oleivorans* KNUC7074 *and R. massiliae* KNUC7586	Pepper (*Capsicum annum* L.)	[160]
		Decreased Na^+^ concentrations in plants.	*B. pumilus*	Rice (*Oryza sativa* L.)	[156]
	Increased N, Fe, P and Mn uptake	IAA production, phosphate solubilization, siderophore production by bacteria.	*Streptomyces* sp.	Wheat (*Triticum aestivum* L.)	[165]
